# Libertinism and Marriage

**Published:** 1902-05

**Authors:** 


### BOOK REVIEWS.

LIBERTINISM AND MARRIAGE. By DR. LOUIS JULLIEN (Paris), Surgeon of Saint-Lazare Prison; Laureate of the Institute, of the Academy of Medicine, and of the Faculty of Medicine of Paris. Translated by R. B. Douglas. Pp. v.—169. Philadelphia: F. A. Davis & Co. 1901. [Price, cloth, $1.00 net.]

A man with Jullien's experience cannot fail to be interesting when he writes, especially when he puts his thoughts on paper in narrative form, touched with anecdote and trimmed with the quaint simile of the French. The book is not a text-book, it does not teach much of anything of treatment, but it tells a great deal which one should know about the management of venereal disease, provided, of course, one is practising where husbands keep mistresses and wives have lovers. Jullien does not mince matters. He speaks out bluntly and incisively. He takes up the questions involved in regular order and treats them in a clear-sighted manner and in language so simple and unscientific that it is all quite refreshing. His entire talk,—the whole thing is much like a confidential chat,—is so free from the affectation of modesty, that one marvels somewhat until he remembers that the incomparable Jullien is in Paris where people live for pleasure and pleasure never dies. If an American had written this book he would have said that a bachelor on the eve of his marriage sometimes, under the influence of the conviviality of a farewell supper, laid himself open to infection. An American would for the sake of modesty, which in medicine at least is a golden virtue, have left something for the imagination and his book would have been entirely free from shocks. Does Jullien? Listen:

"The farewell to bachelor life usually involves, besides a great deal of drinking, certain dangerous proceedings which are apt to favor an attack of blenorrhea. Before breaking off with those pretty girls whom the public—which is not always so just in its appreciation—calls impure women, the young man visits them for the last time, invites them to a farewell spread and—too often—goes to bed with them."

That is the style of the book from beginning to end. It is a genuine heart to heart talk which has all the naïvete, all the unconscious charm, of an "Aunt Mary's Corner" in a young lady's journal.

And in addition to the delights of Jullien's style of writing the book is really valuable, for this great specialist has drawn freely on his experience for material.

He begins with some very plain talk on professional discretion. Then follow chapters on the evolution of gonorrhea—a word he never uses, calling it blenorrhea, which is more polite—acute and chronic, in man; cured in man; acute and chronic and cured in woman. Each chapter is subdivided into three parts: "In Single Life," "Just Before Marriage" and "After Marriage." It is all according to the viewpoint of the reader which chapter will interest him most. If one, deeply imbued with a cleanly desire to add to his scientific knowledge, takes up the book he will in all likehood find most interest in those portions which tell, in the graphic word paintings of a Dickens, the terrible effects of gonorrhea in the innocent; there are those who enjoy a certain style of old and classic writing. They will find here the suggestiveness of a Boccacio, the bluntness of a Smollett and the brilliant frankness of a Rabellais.

The book is valuable in spite of its evident rhetorrhea for there are many suggestions which will not be amiss in the most correct and best regulated practice. It's worth reading anyway, if for no other reason than to learn how a great specialist and diplomat handles cases, and safely steers the ship of domestic happiness through a sea of gonorrheal pus and keeps it from going to pieces on the rocks of divorce. N. W. W.

